# Development and Analytical Validation of a Multiplex Real-Time RT-PCR Assay for Simultaneous Detection of Avian Influenza A Virus and Newcastle Disease Virus

**DOI:** 10.3390/pathogens15080802

**Published:** 2026-07-29

**Authors:** Yerbol Burashev, Saken Khaidarov, Nurdos A. Aubakir, Zamira D. Omarova, Ali B. Tulendibayev, Takhmina U. Argimbayeva, Tangat T. Yermekbay, Khairulla B. Abeuov, Rashida A. Rystaeva, Kulyaisan T. Sultankulova, Sabit K. Kokanov, Nurlan S. Kozhabergenov, Gaukhar O. Shynybekova, Sergazy Sh. Nurabayev, Kuandyk Zhugunissov, Mukhit B. Orynbayev

**Affiliations:** 1 Research Institute for Biological Safety Problems, National Holding QazBioPharm, Ministry of Health Care of the Republic of Kazakhstan, Gvardeiskiy 080409, Kazakhstan; n.aubakir@biosafety.kz (N.A.A.); z.omarova@biosafety.kz (Z.D.O.); a.tulendibayev@biosafety.kz (A.B.T.); t.argimbayeva@biosafety.kz (T.U.A.); t.yermekbai@biosafety.kz (T.T.Y.); kh.abeuov@biosafety.kz (K.B.A.); r.ausekanovna@biosafety.kz (R.A.R.); k.sultankulova@biosafety.kz (K.T.S.); s.kokanov@biosafety.kz (S.K.K.); n.kozhabergenov@biosafety.kz (N.S.K.); g.shynybekova@biosafety.kz (G.O.S.); s.nurabayev@biosafety.kz (S.S.N.); k.zhugunisov@biosafety.kz (K.Z.); omb65@mail.ru (M.B.O.); 2 Department of Molecular Biology and Medical Genetics, Kazakh National Medical University named after S.D. Asfendiyarov, Zheltoksan 37A Street, Almaty 050012, Kazakhstan; 3 Institute of Biotechnology, National Academy of Sciences of the Kyrgyz Republic, Bishkek 720071, Kyrgyzstan; 4 Research and Production Center “MVA Group”, Village Koksay, Karasai District, Almaty 040921, Kazakhstan

**Keywords:** avian influenza A virus, Newcastle disease virus, multiplex real-time RT-PCR, molecular diagnostics, analytical validation, limit of detection, in silico specificity, poultry, One Health, pathogen surveillance

## Abstract

Avian influenza A virus (AIV) and Newcastle disease virus (NDV) are among the most damaging pathogens of poultry, and AIV also carries zoonotic potential that places it within the One Health agenda. The two infections frequently produce overlapping clinical signs, so a method that detects both in a single reaction has clear practical value for surveillance. We developed a duplex one-step real-time reverse-transcription PCR (RT-qPCR) that detects AIV in the FAM channel and NDV in the ROX channel. Primers and hydrolysis probes were designed against conserved regions of the AIV nucleoprotein and matrix genes and the NDV matrix, phosphoprotein and nucleoprotein genes, after alignment of 500 sequences per virus retrieved from GenBank from different geographic regions. Annealing temperature, primer and probe concentrations were optimised, and recombinant plasmids carrying the target fragments served both as positive controls and as quantitative standards. The assay was specific for AIV and NDV, with no cross-reactivity with infectious bronchitis virus and no signal crossover between channels; its analytical sensitivity reached approximately 10–20 RNA copies per reaction. Qualitative results on reference isolates were fully concordant with two commercial veterinary kits (Cohen’s κ = 1.00), and the assay passed inter-laboratory commission testing. This study establishes the design and core analytical performance of a laboratory-developed duplex assay; expanded in silico inclusivity on contemporary sequences, a broader specificity panel, inhibitor-containing matrices, an internal amplification control, and prospective clinical validation are required before routine surveillance deployment.

## 1. Introduction

Avian influenza A virus (AIV), a member of the family Orthomyxoviridae, infects a wide range of domestic and wild birds. Highly pathogenic strains cause severe systemic disease with mortality that can approach 100% in chickens, while low-pathogenic strains often circulate with little or no clinical signs. Several lineages, most prominently the H5 clade 2.3.4.4b viruses, have spread across continents and repeatedly crossed into wild and domestic mammals and, occasionally, humans, which is why AIV sits at the centre of the One Health concept [1,2]. Effective control depends on detecting the virus early, before it amplifies within a flock and spreads to neighbouring premises.

Newcastle disease, caused by virulent strains of Newcastle disease virus (NDV; avian orthoavulavirus 1, family Paramyxoviridae), is one of the most economically important diseases of poultry worldwide [3,4,5]. Velogenic strains can kill an entire unvaccinated flock within days, and the disease remains endemic across much of Africa, Asia and the Middle East despite extensive vaccination. The genetic diversity of class II genotypes continues to expand, which can erode the performance of assays designed against older sequences and creates a recurring need to confirm that diagnostic targets remain conserved [6].

AIV and NDV co-circulate in poultry populations and produce strikingly similar clinical and epidemiological pictures, so the two are difficult to tell apart on clinical grounds alone. Virus isolation in embryonated chicken eggs remains the reference standard, but it takes several days and requires facilities for handling live virus [2,5]. Real-time RT-PCR has largely displaced isolation for first-line screening because it is fast, sensitive and specific, and the assays of Spackman and of Wise, directed at the conserved matrix genes of the two viruses, are still the backbone of routine testing in many laboratories [7,8,9,10]. Combining both targets in one multiplex reaction is an obvious extension: it halves the number of reactions, saves template and reagents, and allows a sample to be screened for both pathogens at once [11,12].

Kazakhstan lies beneath major migratory flyways that connect breeding grounds in northern Eurasia with wintering sites in South Asia and Africa, and incursions of AIV through wild birds are a recurring risk to the national poultry sector; recent high-pathogenicity H5N1 clade 2.3.4.4b outbreaks among wild swans on the Caspian coast illustrate this exposure. Surveillance under these conditions benefits from diagnostic tools that have been developed and analytically validated locally and that do not depend entirely on imported commercial kits, which are costly and not always available. This study describes the development and analytical validation of a duplex real-time RT-PCR for the simultaneous detection of AIV and NDV. We cover the selection of conserved gene targets, in silico specificity assessment, optimisation of the reaction, analytical specificity, the limit of detection, concordance with two commercial reference kits supported by formal statistical analysis, and inter-laboratory commission testing. Consistent with WOAH and MIQE expectations, the work is presented as the development and analytical validation of a laboratory-developed assay, with clinical field validation defined as the necessary next stage rather than claimed here [2,5,13].

## 2. Materials and Methods

### 2.1. Viruses and Reference Materials

Seven AIV isolates (“1102”, “1105”, “486/05”, “489/13”, “491/13”, “492/15” and “495/13”) spanning seven haemagglutinin subtypes (H1N1, H2N2, H3N8, H4N6, H6N2, H7N1 and H13N6; see Table 3 for full strain designations); five NDV (avian orthoavulavirus 1, PMV-1) isolates (“331/14”, “481/15”, “Б-04”, “2698” and “4998”); and two avian paramyxovirus cross-reactivity control isolates (PMV-6/common pochard/Balkhash/5842/2013 and PMV-2/chicken/California/Yucaipa/59; see Table 4) were obtained from the “Collection of Microorganisms” of the Research Institute for Biological Safety Problems (RIBSP). Infectious bronchitis virus (IBV) vaccine strains “Winterfield” and “Kentucky” were included as cross-reactivity controls for a more distantly related avian pathogen. All work with infectious material followed the institute’s biosafety procedures under the containment level appropriate to each isolate.

### 2.2. Sequence Retrieval, Alignment and Oligonucleotide Design

Nucleotide sequences were retrieved from GenBank (NCBI; http://www.ncbi.nlm.nih.gov/genbank, accessed on 1 July 2026). For each virus, 500 isolate sequences from different geographic regions were collected and aligned in MEGA version 10. Conserved regions were identified in the nucleoprotein (NP) and matrix (M) genes of AIV and in the matrix (M), phosphoprotein (P) and nucleoprotein (NP) genes of NDV; these genes carry sequence stretches that are specific to the target virus yet conserved across isolates.

Primers and hydrolysis probes were designed within these regions using the CLC Genomics Workbench module version 12 (Qiagen, Hilden, Germany), with attention to primer length, melting temperature, GC content, complementarity, target and primer secondary structure, and the likelihood of homo- and heterodimer formation. AIV probes were labelled with FAM and quenched with BHQ-1; NDV probes were labelled with ROX and quenched with BHQ-2, a pairing chosen to keep the two reporters spectrally distinct in a single tube. Candidate oligonucleotides were checked for specificity by BLAST against the GenBank database, and only those returning 100% identity with the intended target were retained. In total, 52 primers and 6 probes were synthesised and screened, and the most efficient sets were carried forward. All oligonucleotides were synthesised by DNK-Sintez (Moscow, Russia) and reconstituted to working stocks of 10 pmol/µL.

### 2.3. RNA Extraction

Viral RNA was extracted with the QIAamp Viral RNA Mini Kit (Qiagen, Hilden, Germany) according to the manufacturer’s spin-column protocol. RNA concentration and purity were measured on a NanoDrop 2000 spectrophotometer (NanoDrop Technologies, Wilmington, DE, USA). Extractions were carried out in a biosafety cabinet under the containment level appropriate to the isolate.

### 2.4. Recombinant Plasmid Controls and Copy-Number Calculation

To generate reproducible positive controls and copy-number standards, target fragments of the AIV NP gene and the NDV M gene were amplified and cloned into the pGEM-T Easy Vector (Promega, Madison, WI, USA; 3015 bp without insert). Ligation products were used to transform competent Escherichia coli by chemical transformation; recombinant clones were identified by blue–white selection, grown in LB broth at 37 °C for 16 h, and plasmids were recovered by alkaline lysis. The final constructs measured 3115 bp (AIV NP insert, ≈100 bp) and 3242 bp (NDV M insert, ≈227 bp).

Plasmid copy number was calculated from the spectrophotometrically determined DNA concentration and the plasmid length (no digital-PCR calibration was used) with the formula: copies/µL = (concentration [ng/µL] × 6.022 × 10^23^) / (length [bp] × 660 × 10^9^). On this basis, 2–5 ag of plasmid corresponds to a single target molecule. Ten-fold serial dilutions of the quantified standards were prepared and used for the limit-of-detection experiments (Section 2.9).

### 2.5. One-Step RT-qPCR Chemistry and Reaction Composition

One-step chemistries were used throughout: the SuperScript III One-Step RT-PCR System with Platinum Taq DNA Polymerase (Invitrogen, Carlsbad, CA, USA) and the One Step RT-PCR Kit (Qiagen, Hilden, Germany). The supplied enzyme mixture provides both reverse-transcriptase and DNA-polymerase activity. The final duplex reaction volume was 25 µL and contained 5 µL of 5× reaction buffer, 1 µL enzyme mix, 1 µL of 10 mM dNTP mix, 1.25 µL of each 10 µM primer (≈500 nM final), 1 µL of each 5 µM hydrolysis probe (≈200 nM final), 1 µL template RNA, and nuclease-free water to volume. Mg^2+^ was supplied by the commercial reaction buffer and was not adjusted separately. Reporting followed the MIQE recommendations for quantitative PCR [13].

### 2.6. Instrument and Fluorescence Acquisition

Endpoint gradient RT-PCR for primer screening was run on a ProFlex PCR System (Applied Biosystems, Foster City, CA, USA); products were resolved on 2% agarose gels in TAE buffer at 400 mA with SYBR Safe DNA gel stain (Invitrogen, Carlsbad, CA, USA), sized against a 50 bp DNA ladder (Invitrogen, Carlsbad, CA, USA), and imaged on a Bio-Rad gel-documentation system (Bio-Rad, Hercules, CA, USA). Real-time RT-PCR was performed on a Rotor-Gene Q instrument (Qiagen, Hilden, Germany) and analysed in Rotor-Gene Q software v1.8.187.5. Fluorescence was acquired once per cycle during the combined annealing/extension step (55 °C, 25 s), with AIV read in the FAM/Green channel and NDV in the ROX/Orange channel.

### 2.7. Optimisation of Reaction Parameters

The annealing temperature was optimised by gradient RT-PCR at 50, 52.5, 55, 57.5, 60 and 62.5 °C using the standard component ratios of the commercial kit and a primer concentration of 400 nM. For endpoint gradient screening, cycling comprised reverse transcription at 55 °C for 15 min and initial denaturation at 94 °C for 2 min, followed by 40 cycles of denaturation at 94 °C for 15 s, annealing for 20 s across the temperature gradient, and elongation at 68 °C for 45 s, with a final elongation at 68 °C for 7 min. Primer concentrations were then tested from 0.1 to 0.8 µM—spanning the 0.2–0.45 µM range typical of real-time RT-PCR—and probe concentrations from 50 to 250 nM, with the remaining components held constant.

### 2.8. Analytical Specificity

Specificity was assessed empirically against RNA from NDV, AIV, IBV, and two additional avian paramyxoviruses (PMV-6/common pochard/Balkhash/5842/2013 and PMV-2/chicken/California/Yucaipa/59). Cloned plasmid DNA carrying the NDV M-gene and AIV NP-gene fragments served as positive controls, and nuclease-free, deionized water served as the no-template control. Amplification in the FAM channel was scored for AIV and in the ROX channel for NDV. Empirical specificity was complemented by the in silico BLAST analysis described in Section 2.2, in which every primer and probe matched only its intended target.

### 2.9. Analytical Sensitivity

The limit of detection was determined with ten-fold serial dilutions of the plasmid standards, spanning 2 ng to 2 ag per reaction, tested under optimised conditions. Reproducible amplification was defined as a typical sigmoidal amplification curve with Ct below the assay cut-off; the lowest dilution yielding consistent amplification was taken as the analytical sensitivity, while detection at only the very lowest copy number was recorded as inconsistent (±).

### 2.10. Comparison with Commercial Kits and Statistical Analysis

To benchmark the assay, the same RNA panels were tested with two commercial veterinary kits: PCR-GRIPP-A-FACTOR (cat. no. R10515-VET) for AIV and PCR-NEWCASTLE-FACTOR (cat. no. R15418-VET) for NDV, each used according to its instructions. Qualitative agreement was evaluated as overall, positive and negative percent agreement and Cohen’s κ. For paired Ct values, Pearson correlation, mean Ct difference, a paired *t*-test, and Bland–Altman limits of agreement were calculated. Because the isolate panel was small, these comparisons were interpreted as preliminary method-comparison data rather than definitive diagnostic-performance estimates. Calculations were performed in standard statistical software, with *p* < 0.05 considered significant.

### 2.11. Normative-Technical Documentation and Inter-Laboratory Commission Testing

The assay was formalised as a laboratory test system, and a full set of normative-technical documentation was prepared: a manufacturing and quality-control instruction, an application (user) guideline, and a Standard of the Organisation (“Test system for the detection of avian influenza A virus and Newcastle disease virus RNA by multiplex real-time RT-PCR”; No. ST-RIBSP-061040004937-016-2025). The test system was then submitted to inter-laboratory commission testing at RIBSP, in which specificity and sensitivity were re-examined by an independent panel on coded samples and checked against the parameters defined in this documentation.

## 3. Results

### 3.1. Design of Primers and Probes Targeting Conserved Regions

Alignment of 500 sequences per virus resolved several conserved blocks suitable for primer and probe placement. For AIV, these lay in the NP and M genes, and for NDV in the M, P and NP genes. After BLAST filtering and screening of all 52 primers and six probes, the sets listed in Table 1 were selected. The AIV targets yield amplicons of 79 bp (M), 100 bp (NP) and 319 bp (NP2) and are read in the FAM channel; the NDV targets yield amplicons of 227 bp (M), 160 bp (P) and 485 bp (NP) and are read in the ROX channel. The NP-gene fragment of AIV and the M-gene fragment of NDV were cloned into plasmid controls and used for quantitation.

### 3.2. Optimisation of the Annealing Temperature

**Figure 1:**
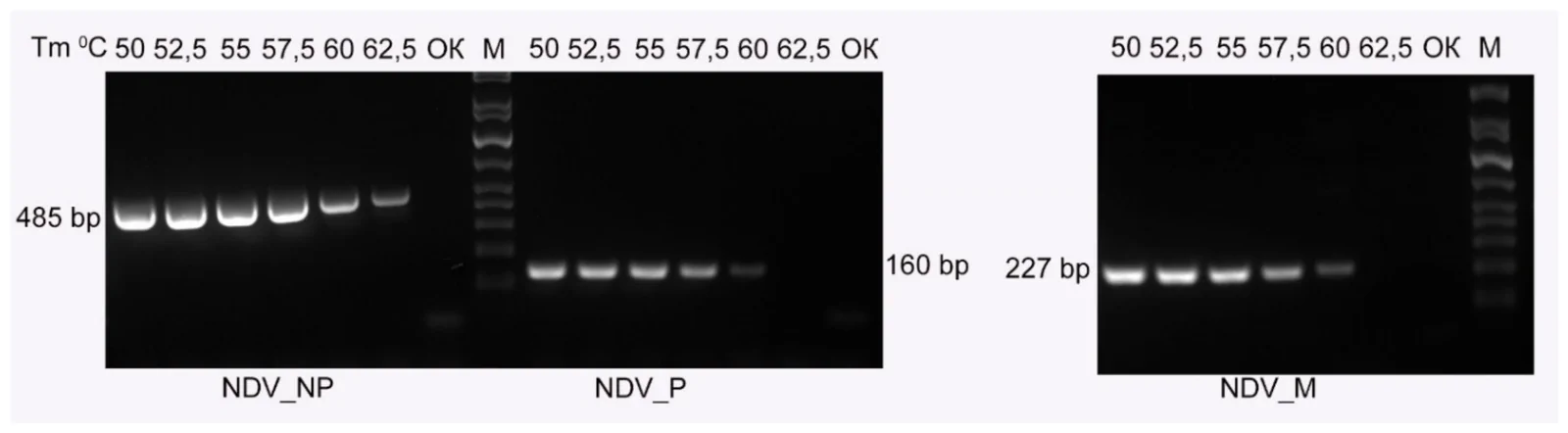
Gradient RT-PCR for the Newcastle disease virus primer sets (NDV_NP, NDV_P, NDV_M) across annealing temperatures of 50–62.5 °C. M, DNA molecular weight marker (50 bp ladder, Invitrogen); OK, positive control.

**Figure 2:**
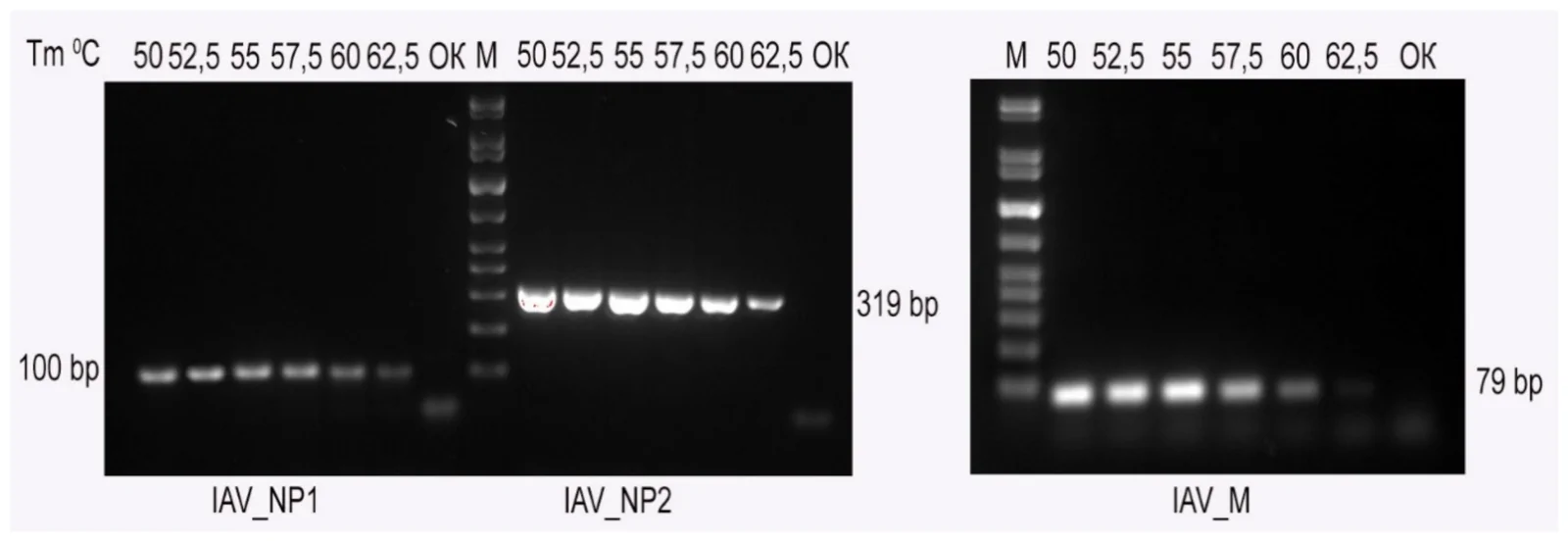
Gradient RT-PCR for the avian influenza A virus primer sets (AIV_NP1, AIV_NP2, AIV_M) across annealing temperatures of 50–62.5 °C. M, DNA molecular weight marker (50 bp ladder, Invitrogen); OK, positive control.

Gradient RT-PCR showed clear temperature dependence. For NDV, the NP set amplified across the whole gradient but most efficiently between 50 and 57.5 °C, whereas the P and M sets failed at 62.5 °C and performed poorly at 60 °C, with an effective window of 50–55 °C (Figure 1). The AIV sets (M, NP, and NP2) behaved similarly: amplification was weak or absent at 60 and 62.5 °C, and all three worked efficiently at 55 °C (Figure 2). A common annealing temperature of 55 °C was therefore adopted for the duplex reaction, as it supports robust amplification of all targets in a single tube.

### 3.3. Optimisation of Primer and Probe Concentrations

**Figure 3:**
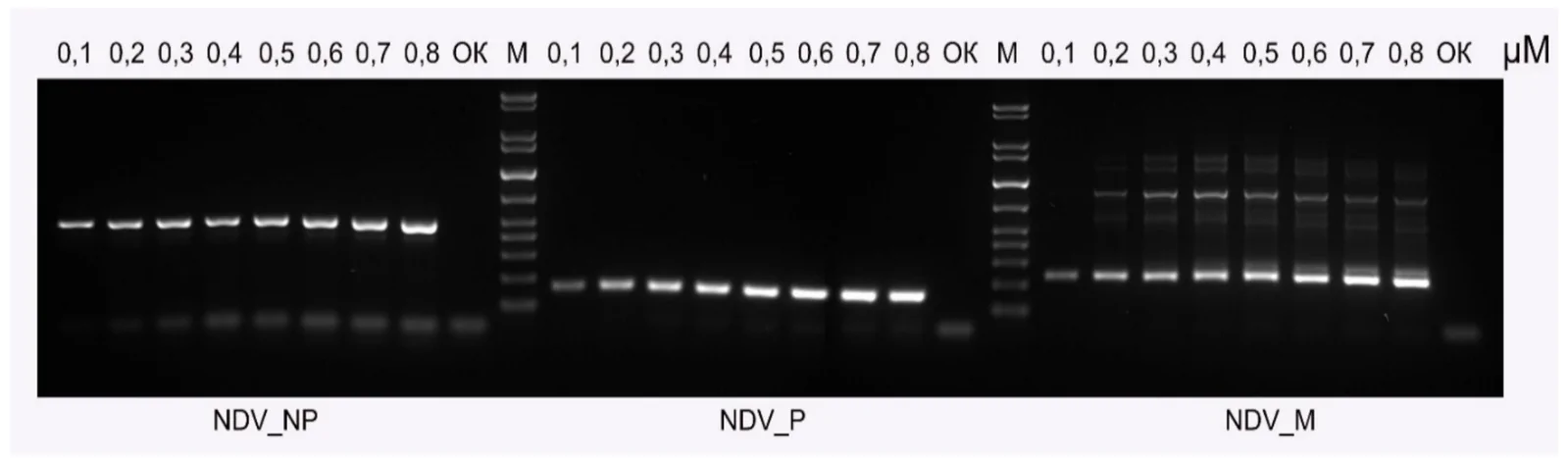
Optimisation of primer concentrations for the NDV_NP, NDV_P and NDV_M sets. M, DNA molecular weight marker (50 bp ladder, Invitrogen); OK, positive control.

**Figure 4:**
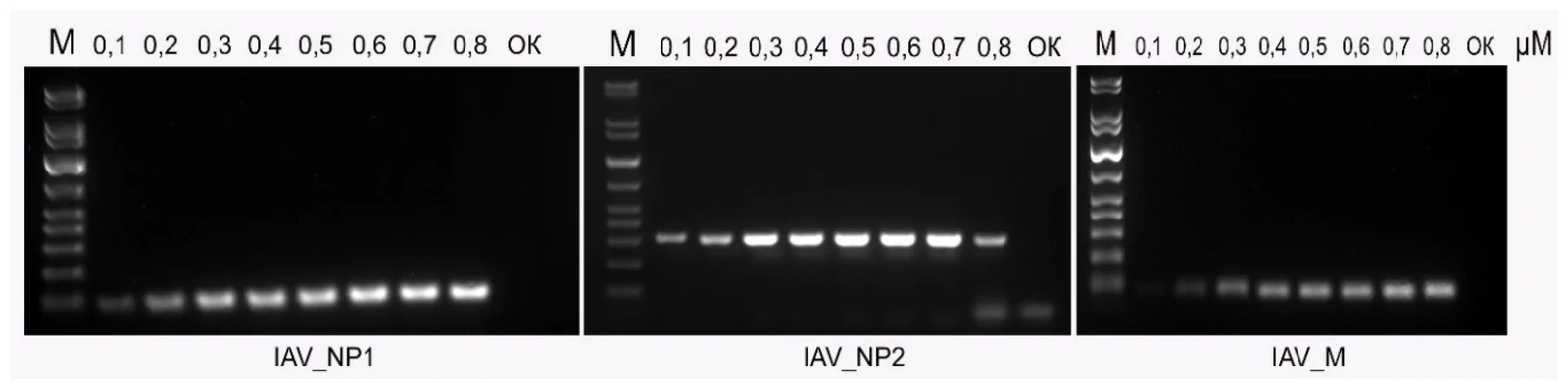
Optimisation of primer concentrations for the AIV_NP1, AIV_NP2 and AIV_M sets. M, DNA molecular weight marker (50 bp ladder, Invitrogen); OK, positive control.

**Figure 5:**
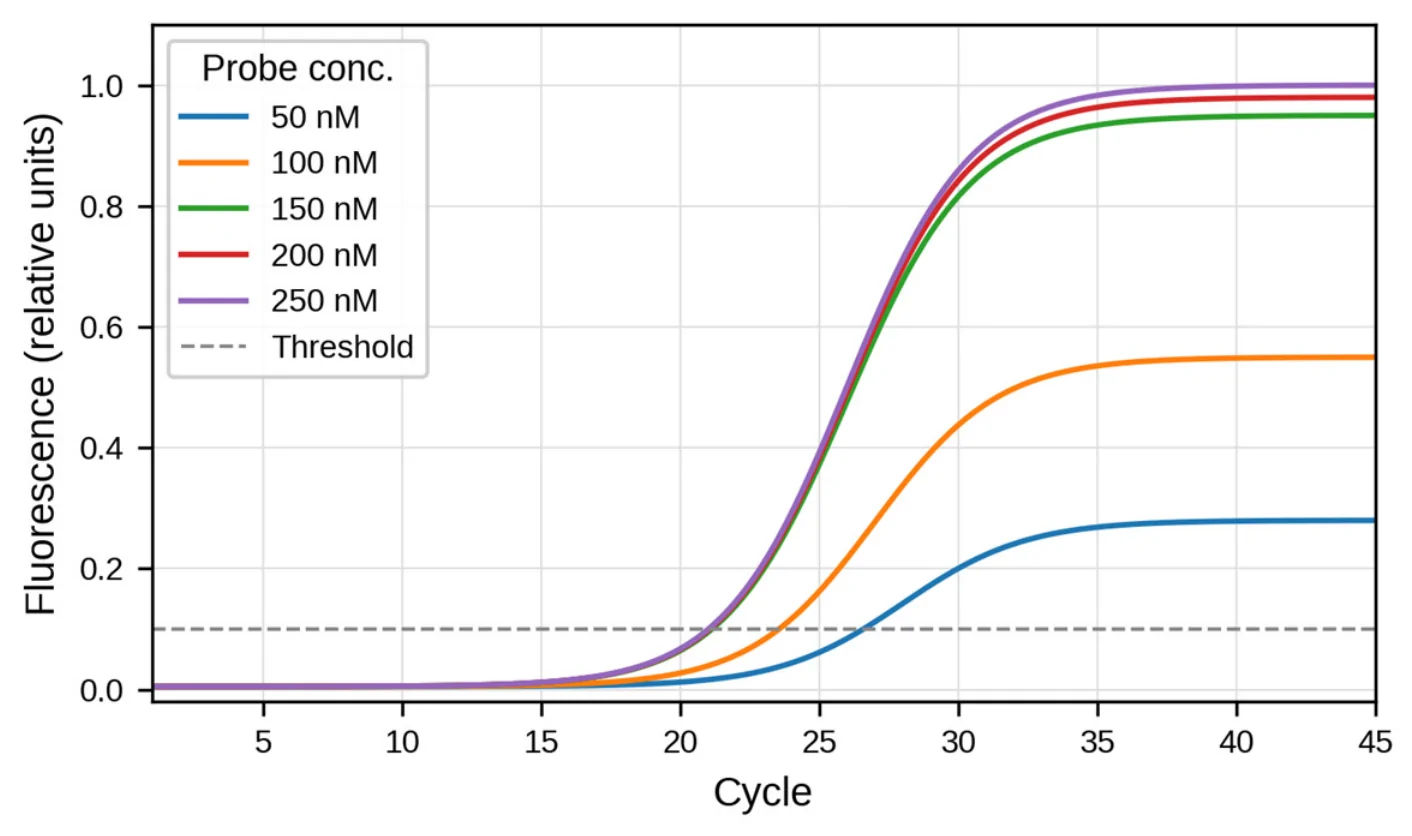
Optimisation of hydrolysis probe concentrations (50–250 nM); fluorescence plateaued at 150–250 nM.

Across the 0.1–0.8 µM range, every primer concentration produced specific product, but the strongest, most consistent amplicons appeared at 300 nM and above; working concentrations of 400–600 nM were selected (Figure 3 and Figure 4). For the hydrolysis probes, fluorescence increased with concentration up to a plateau, and all probes performed well between 150 and 250 nM (Figure 5). Based on these results, the final reaction used ≈500 nM of each primer and ≈200 nM of each probe. The complete thermal programme is given in Table 2.

### 3.4. Analytical Specificity

**Figure 6:**
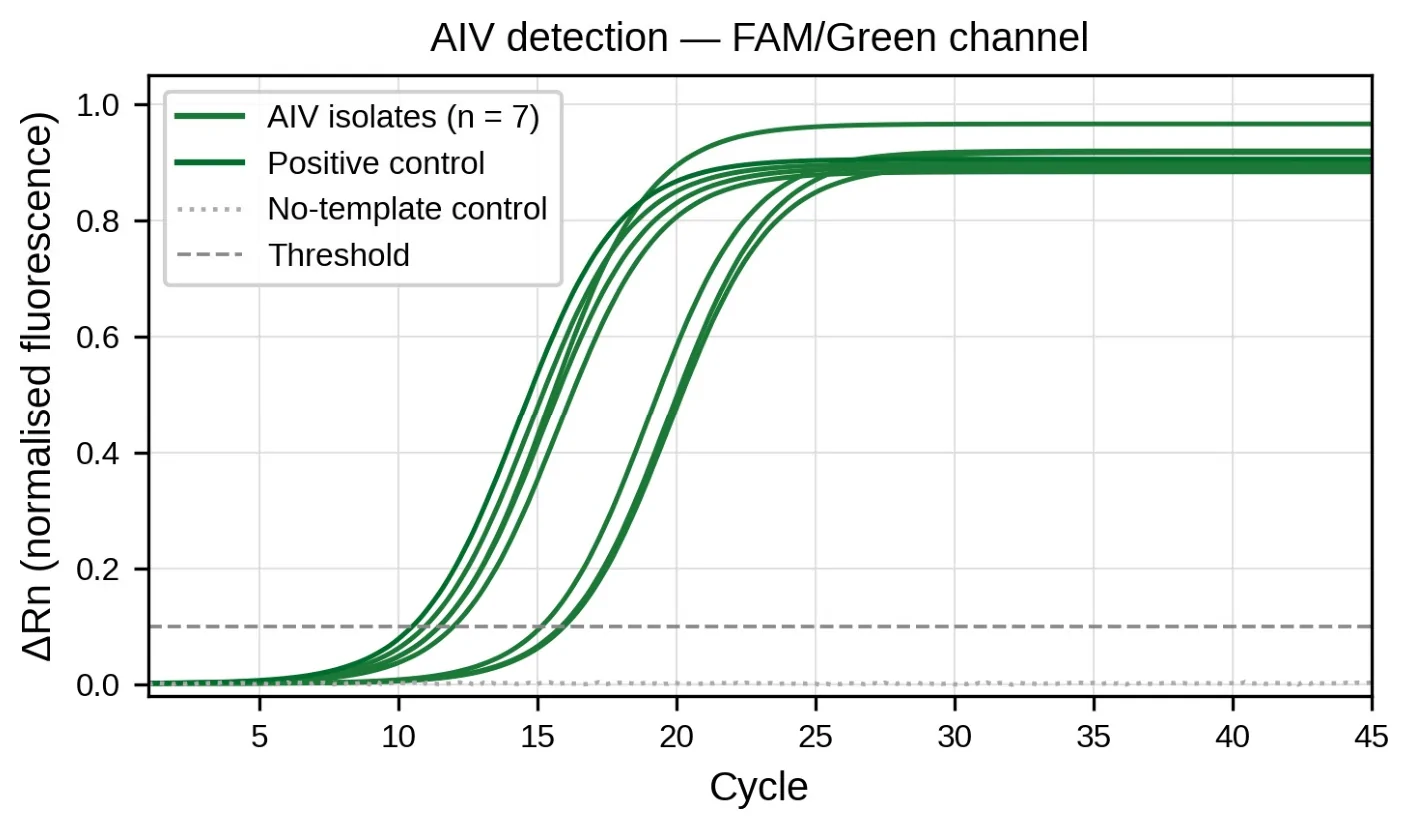
Real-time RT-PCR amplification of AIV reference isolates and controls in the FAM (green) channel.

**Figure 7:**
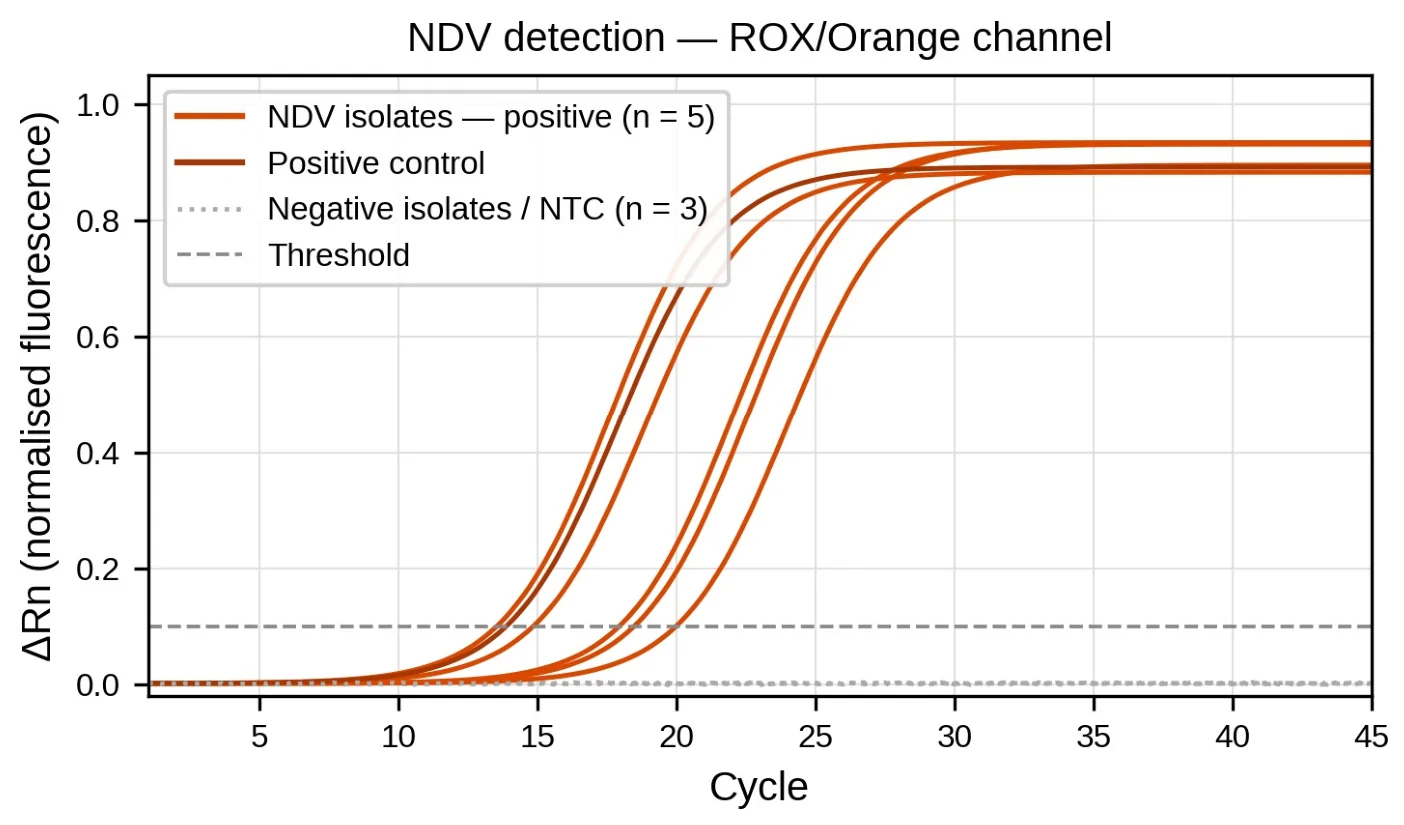
Real-time RT-PCR amplification of NDV reference isolates and controls in the ROX (orange) channel.

Under the optimised conditions, the duplex assay discriminated the two viruses cleanly. AIV reference isolates generated signals only in the FAM channel, with Ct values between roughly 14.8 and 19.8, and gave no signal in the ROX channel. NDV reference isolates generated signals only in the ROX channel, with Ct values between roughly 17.6 and 24.0, and were negative in the FAM channel. The IBV strains “Winterfield” and “Kentucky” produced no amplification in either channel, and the no-template control remained flat throughout (Figure 6 and Figure 7). The plasmid positive controls amplified in their expected channels. In silico, every primer and probe matched only its intended target by BLAST. The assay therefore detects AIV and NDV without cross-reacting with IBV and without crossover between reporters; the broader avian-pathogen panel needed to fully exclude field cross-reactivity is addressed in the Discussion.

### 3.5. Concordance with Commercial Reference Kits and Statistical Agreement

The same RNA panels were tested in parallel with the developed duplex assay and with the corresponding commercial veterinary kits. For AIV, all seven isolates were positive by both methods, and the Ct values tracked closely (Table 3). For NDV, five of seven isolates were positive by both methods; isolates “485/11” and “955/15” were negative by both the developed assay and the commercial NDV kit (Table 4). Because the two negatives were reproduced by an independent commercial assay, they reflect the samples themselves—PMV-6 and PMV-2, which are distinct avian paramyxovirus serotypes and are not detected by the NDV-specific primers and probes by design—rather than a failure of the new assay.

Qualitative results were fully concordant with both commercial kits (overall, positive and negative percent agreement 100%; Cohen’s κ = 1.00; Table 5). For AIV, paired Ct values correlated strongly (Pearson *r* = 0.97, *p* < 0.001), with a mean difference of 0.83 ± 0.67 cycles (paired *t* = 3.29, *p* = 0.017) and Bland–Altman limits of agreement of −0.48 to 2.14 cycles; the developed assay read marginally higher Ct values, a systematic offset below one cycle that did not affect qualitative interpretation. For NDV, the five quantifiable pairs were qualitatively concordant (mean difference 1.52 ± 2.44 cycles; paired t = 1.40, *p* = 0.234; Bland–Altman −3.25 to 6.30 cycles) but too few for a robust correlation estimate. Owing to the limited panel size, these comparisons are reported as preliminary; categorical concordance (κ = 1.00) is the primary result.

### 3.6. Analytical Sensitivity

**Figure 8:**
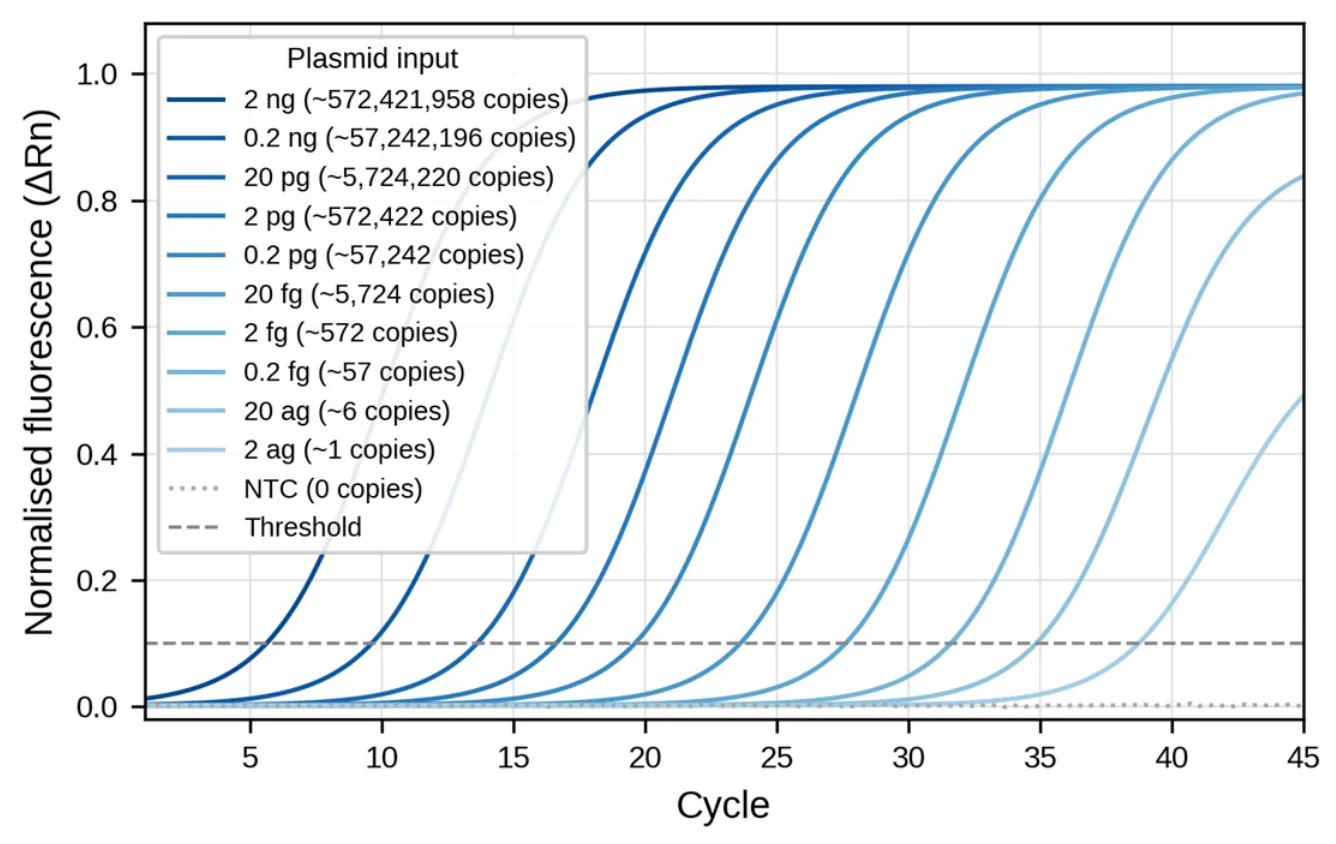
Amplification curves for the plasmid-standard dilution series used to determine analytical sensitivity.

Ten-fold dilution series of the plasmid standards were used to establish the limit of detection (Table 6). Reproducible amplification extended down to the 20 ag dilutions, equivalent to roughly six target copies, with occasional detection at 2 ag (≈1 copy). On this basis, the analytical sensitivity of the duplex assay is approximately 10–20 RNA copies per reaction for both AIV and NDV (Figure 8), which is within the range expected of diagnostic-grade real-time assays.

### 3.7. Normative-Technical Documentation and Inter-Laboratory Commission Testing

The assay was evaluated by an independent commission at RIBSP on coded RNA samples. It correctly identified the AIV and NDV samples, gave no false-positive results, and reproduced the analytical sensitivity of approximately 10–20 copies per reaction observed in development. The commission concluded that the duplex assay performs specifically and with a sensitivity adequate for laboratory diagnostic use.

The assay was formalised as a laboratory test system supported by a complete set of normative-technical documentation—a manufacturing and quality-control instruction, an application guideline, and a Standard of the Organisation (No. ST-RIBSP-061040004937-016-2025). In the commission trials, the specificity and sensitivity of the test system met the parameters defined in this documentation, providing an institutional quality-assurance basis for its subsequent clinical validation.

## 4. Discussion

We set out to build a single-tube assay that reports AIV and NDV separately, and the duplex RT-qPCR does exactly that: AIV in the FAM channel, NDV in the ROX channel, from one aliquot of RNA. The practical appeal is straightforward. Two of the most consequential poultry viruses present alike in the field, and being able to rule both in or out in one run shortens the path from a sick flock to a laboratory answer. We frame the present work, however, as the development and analytical validation of a laboratory-developed assay rather than as a completed field-surveillance tool, consistent with WOAH guidance that molecular methods be shown fit for purpose on clinical material before deployment [2,5].

The choice of targets follows established practice while broadening the sequence base. The matrix gene of type A influenza is the most conserved coding region of the genome and has anchored AIV screening since the assays of Fouchier and of Spackman [7,8]; the matrix gene of NDV plays the same role in the Wise assay that underpins much of routine ND diagnosis [9]. Designing against 500 aligned sequences per virus from diverse geographic regions, and retaining only oligonucleotides with full BLAST identity to the target, was a deliberate hedge against the genetic drift that has eroded older NDV assays as new class II genotypes emerged [6]. For NDV in particular, the duplex serves as a screening assay, while pathotype/virulence assignment requires sequencing of the fusion-protein cleavage site. Pairing FAM with ROX, rather than two spectrally adjacent dyes, kept the channels clean enough that no crossover was seen even at high template loads.

Analytical sensitivity of about 10–20 copies per reaction sits comfortably alongside published multiplex assays for the same viruses. The duplex NDV/H5-AIV assay of Zhang and colleagues reported a limit of five copies per reaction with intra-/inter-assay CV below 1%, and a triplex AIV/NDV/duck Tembusu assay reported about ten copies per microlitre with intra-/inter-day relative standard deviation below 4.44% [11,12]; our figures are in the same order of magnitude and are sufficient to catch infections well before clinical breakdown. Relative to those systems, the present assay’s strengths are a simple two-channel readout with separate AIV and NDV detection, validation on locally available reference isolates, and plasmid-based quantitative controls. Its current limitations relative to some published systems are a clinical-sample panel not yet sufficient for full diagnostic validation, a limited cross-reactivity panel, the absence of AIV subtyping and NDV pathotyping, and the absence of an internal amplification control—the items that define the next development stage.

The most persuasive part of the validation, to our mind, is not the dilution series but the agreement with the commercial kits, including the two NDV samples that both methods scored as negative. When an independent assay reproduces your negatives as well as your positives, the result is much harder to dismiss as an artefact of primer design. We report this agreement with formal statistics (Cohen’s κ = 1.00; Pearson correlation and Bland–Altman analysis) and note honestly the sub-cycle AIV offset rather than smoothing it over; given the small panel, these are preliminary method-comparison data, and Ct correlation is secondary to categorical concordance.

Several limitations follow directly from the study design and have shaped the moderated conclusions. First, the analytical-specificity panel comprised AIV, NDV, and two IBV vaccine strains; this confirms the absence of IBV cross-reactivity and of channel crossover but does not exclude cross-reactivity with all avian respiratory pathogens. A broader panel—avian metapneumovirus, infectious laryngotracheitis virus, avian reovirus, infectious bursal disease virus, fowl adenovirus, other avian avulaviruses, and relevant bacterial agents of the respiratory disease complex—should be included in future validation. Second, the present validation did not include an inhibition study with mucus, blood, faecal or tissue-homogenate matrices, so matrix-associated inhibition remains to be assessed. Third, validation used plasmid standards and archived reference isolates rather than a prospective clinical panel; diagnostic sensitivity, diagnostic specificity and performance in complex field matrices therefore remain to be established. A prospective clinical panel—spanning oropharyngeal and cloacal swabs and organ homogenates from several bird species across low, intermediate and high viral loads, benchmarked against virus isolation and/or sequence confirmation—is the required next step.

The single most actionable improvement for routine reliability is an internal amplification control. WOAH notes that an internal positive control verifies a valid run and that PCR inhibitors in some matrices can cause false-negative results [2]. The current version does not include such a control; the next version of the test system will incorporate an exogenous heterologous RNA internal control added before extraction, so that both extraction efficiency and amplification inhibition are monitored, run as a parallel reaction on the same extract where a free optical channel is unavailable.

From a workflow perspective, the duplex format screens for AIV and NDV in a single reaction, halving the reactions, template and reagents needed relative to two singleplex assays. With the described thermal profile, amplification takes about 75 min; turnaround from extracted RNA to result is roughly 1.5–2 h, and the full workflow including extraction is about 2–3 h depending on sample number. Throughput is set by the rotor capacity of the instrument. A formal cost-effectiveness analysis was not performed, but the reduced reaction counts and reliance on locally produced controls lower the per-sample cost relative to imported singleplex kits.

For a country positioned under major migratory flyways, a locally developed and analytically validated assay has value beyond the bench. It reduces dependence on imported kits, lowers the per-sample cost of surveillance, and gives national veterinary laboratories a tool they can deploy and troubleshoot themselves once clinical validation is complete. That fits the One Health logic of the disease problem: AIV, in particular, moves between wild birds, poultry, and, increasingly, mammals, and timely differential detection at the poultry interface is part of how spillover is caught early [1,2].

## 5. Conclusions

A duplex one-step real-time RT-PCR assay was developed and analytically validated for the simultaneous detection of avian influenza A virus and Newcastle disease virus. It showed clean channel separation, no cross-reactivity with the IBV strains tested, full qualitative concordance with two commercial kits (Cohen’s κ = 1.00), and an analytical sensitivity of approximately 10–20 copies per reaction with plasmid standards, and it passed inter-laboratory commission testing. The study establishes the assay design and core analytical performance of the test system. Expanded validation—updated in silico inclusivity on contemporary sequences, a broader avian-pathogen specificity panel, inhibitor-containing matrices, an internal amplification control, and a prospective clinical-sample panel—is required before the assay can be recommended for routine poultry surveillance. The assay nonetheless provides a technically validated, locally produced foundation for that next stage, and for future additions such as AIV subtyping and NDV pathotyping.

## Figures and Tables

**Table 1:** Primers and hydrolysis probes designed and selected for the duplex real-time RT-PCR assay.

Virus	Oligo	Sequence (5′–3′)	Tm (°C)	GC (%)	Amplicon (bp)
*Newcastle disease virus*	NDV_M_F	AGARGRAAARATAAGRAGACTC	53–60	27–45	227
	NDV_M_probe	ROX-ATAGCAAATGCYTCYCCYCARGT-BHQ-2	59–67	39–57	
	NDV_M_R	GTRCCDGCYTGAATGATGA	53–60	42–58	
	NDV_P_F	GARCACAGYATATCATGGAC	54–58	40–50	160
	NDV_P_probe	ROX-TCAGCTGGTGYAAYCCYTC-BHQ-2	55–62	47–63	
	NDV_P_R	CCTCCATCATRGACATCAT	53–55	42–47	
	NDV_NP_F	ATTGCTGTTAGYGAGGAYGC	56–60	45–55	485
	NDV_NP_probe	ROX-ATGACTGCRTAYGAGACRGC-BHQ-2	56–63	45–60	
	NDV_NP_R	AGTTGRATTGYACTYCTGCA	52–58	35–50	
*Avian influenza A virus*	AIV_M_F	CTCATGGARTGGCTAAAGAC	56–58	45–50	79
	AIV_M_probe	FAM-ACCRATCYTGTCACCTYTGAC-BHQ-1	57–63	43–57	
	AIV_M_R	ACGGTGAGCGTRAANACRAA	54–60	40–55	
	AIV_NP_F	GTCTTCGAGCTYTCRGACGA	58–63	50–60	100
	AIV_NP_probe	FAM-ATCGTGCCTTCCTTTGACATGA-BHQ-1	60	45	
	AIV_NP_R	TAYTCCTCTGCAYTGTCYCC	56–63	45–60	
	AIV_NP2_F	CACCTGATGATCTGGCATTC	58	50	319
	AIV_NP2_probe	FAM-AATGATGCCACATAYCAGAGRAC-BHQ-1	59–63	39–48	
	AIV_NP2_R	TGRTCCATCATTGCTCTTTG	54–56	40–45	

Degenerate base codes follow IUPAC nomenclature (R = A/G; Y = C/T; D = A/G/T; N = A/C/G/T). FAM and ROX are the reporter dyes; BHQ-1 and BHQ-2 are the corresponding quenchers.

**Table 2:** Thermal program for the duplex real-time RT-PCR assay.

Step	Temperature	Time	Cycles
1	50 °C	30 min	1 (reverse transcription)
2	95 °C	15 min	1 (polymerase activation)
3	94 °C	15 s	45
	55 °C	25 s (FAM and ROX acquisition)	

**Table 3:** Ct values for avian influenza A virus isolates by the developed duplex assay (FAM channel) and the commercial PCR-GRIPP-A-FACTOR kit.

Sample	Developed Assay, Ct	Commercial Kit, Ct
AIV “1102”	19.76	18.78
*H1N1; A/duck/Alberta/35/76*		
AIV “1105”	19.7	18.62
*H2N2; A/silver gull/Atyrau/2186/07*		
AIV “486/05”	19	16.88
*H3N8; A/duck/California/72*		
AIV “489/13”	15.26	15.1
*H4N6; A/duck/Czechoslovakia/56*		
AIV “491/13”	15.42	15.02
*H6N2; A/turkey/Massachusetts/3740/65*		
AIV “492/15”	14.76	14.46
*H7N1; A/chicken/Rostock/29*		
AIV “495/13”	15.75	14.98
*H13N6; A/silver gull/Atyrau/280/02*		
Positive control	14.32	22.53
No-template control	–	–

**Table 4:** Ct values for Newcastle disease virus isolates by the developed duplex assay (ROX channel) and the commercial PCR-NEWCASTLE-FACTOR kit.

Sample	Developed Assay, Ct	Commercial Kit, Ct
NDV “331/14”	22.04	19.16
*PMV-1 (NDV); T-53*		
NDV “481/15”	18.83	17.94
*PMV-1 (NDV); PMV-1/turkey/Zhambyl/536/90*		
NDV “485/11”	Undetermined	Undetermined
*PMV-6 †; avian paramyxovirus 6; common pochard/Balkhash/5842/2013*		
NDV “955/15”	Undetermined	Undetermined
*PMV-2 †; avian paramyxovirus 2; chicken/California/Yucaipa/59*		
NDV “Б-04”	17.64	20.09
*PMV-1 (NDV); RIBSP collection*		
NDV “2698”	24	20.39
*PMV-1 (NDV); Chapai/12/2010*		
NDV “4998”	22.58	19.89
*PMV-1 (NDV); RIBSP collection*		
Positive control	17.88	19.14
No-template control	–	–

† Avian paramyxovirus serotype 6 (PMV-6) and serotype 2 (PMV-2) are distinct from Newcastle disease virus (avian orthoavulavirus 1, PMV-1). These isolates were included as within-family cross-reactivity controls; their negative result confirms the assay does not detect non-NDV avian paramyxovirus serotypes. Undetermined: No fluorescence signal detected within 45 amplification cycles (Rotor-Gene Q output for a negative result).

**Table 5:** Method-comparison statistics between the developed duplex assay and the commercial kits.

Comparison	*n*	Pearson *r* (*p*)	Mean ΔCt ± SD (cycles)	Paired *t* (*p*)
AIV (FAM)	7	0.97 (<0.001)	0.83 ± 0.67	3.29 (0.017)
NDV (ROX)	5	0.41 (0.49)	1.52 ± 2.44	1.40 (0.234)

Bland–Altman limits of agreement: AIV −0.48 to 2.14 cycles; NDV −3.25 to 6.30 cycles. Qualitative agreement: overall/positive/negative percent agreement 100%; Cohen’s κ = 1.00. The wide NDV interval reflects only five quantifiable pairs and is underpowered.

**Table 6:** Detection of ten-fold plasmid-standard dilutions, with estimated target copy number per reaction.

Plasmid Amount	Estimated Copies	Detection
2 ng	572,421,958	+
0.2 ng	57,242,196	+
20 pg	5,724,220	+
2 pg	572,422	+
0.2 pg	57,242	+
20 fg	5724	+
2 fg	572	+
0.2 fg	57	+
20 ag	6	+
2 ag	1	±
No-template control	0	−

+, reproducible amplification; ±, inconsistent amplification near the detection limit; −, no amplification.

## Data Availability

The reference nucleotide sequences used for primer and probe design are available in GenBank (https://www.ncbi.nlm.nih.gov/genbank, accessed on 1 July 2026). The data generated and analysed in this study are available from the corresponding authors on reasonable request.
